# Cleaved CD44 intracellular domain supports activation of stemness factors and promotes tumorigenesis of breast cancer

**DOI:** 10.18632/oncotarget.3325

**Published:** 2015-04-02

**Authors:** Yunhee Cho, Hyun-Woo Lee, Hyeok-Gu Kang, Hye-Young Kim, Seok-Jun Kim, Kyung-Hee Chun

**Affiliations:** ^1^ Department of Biochemistry & Molecular Biology, Yonsei University College of Medicine, Seodaemun-gu, Seoul 120-752, Korea; ^2^ Department of Biochemistry, College of Life Science and Biotechnology, Seodaemun-gu, Seoul 120-752, Korea; ^3^ Brain Korea 21 Plus Project for Medical Science, Yonsei University, Seodaemun-gu, Seoul 120-752, Korea

**Keywords:** CD44, intracellular domain, stemness factors, breast cancer

## Abstract

CD44 plays a role in the progression of tumors and is expressed in cancer stem cells (CSCs). However, the mechanisms underlying the crosstalk of CD44 with stemness genes in CSC maintenance remains unclear. In this study, we demonstrated how the cleaved intracellular domain of CD44 (CD44ICD) activates stemness factors such as Nanog, Sox2 and Oct4, and contributes to the tumorigenesis of breast cancer. We have found that the overexpression of CD44ICD increased mammosphere formation in breast cancer cells. Treatment with a γ-secretase inhibitor (GSI), which blocks the cleavage of CD44ICD, interfered with mammosphere formation. Interestingly, CD44ICD decreased the expression levels and nuclear localization of stemness factors, but overexpression of CD44ICD reversed these effects. In addition, we showed that nuclear localization of CD44ICD is important for transcriptional activation of the stemness factors. Furthermore, CD44ICD-overexpressed cells exhibited strong tumorigenecity and greater metastatic potential than did the control cells or CD44-depleted cells *in vivo* in mice models. Taken together, it was supposed that CD44 promotes tumorigenesis through the interaction and nuclear-translocation of its intracellular domain and stemness factors. We suggest that the prevention of cleavage and nuclear-translocation of CD44ICD is a potential target in treating breast cancer.

## INTRODUCTION

CD44, a receptor that binds to hyaluronic acid (HA), is a multistructural and multifunctional molecule and is responsible for cell-to-cell communication and that between the cells and extracellular matrix (ECM) [1]. CD44 is able to alter tumor environments through its involvement in cell trafficking, lymph node homing, and the coordination of cytokines and growth factor signaling [2]. As a major glycosaminoglycan in the ECM, HA accumulates at sites of cell division and rapid matrix remodeling, as is seen during tumorigenesis. HA activates signaling by binding to CD44 variants (CD44v), which result from the alternate splicing of the *CD44* gene. [3, 4]. However, the intracellular domain was equal to all CD44 variants [5]. The smallest form of CD44, designated standard CD44 (CD44s), is abundantly expressed in both normal and cancer cells, whereas the CD44v, which contain a variable number of exon insertions (v1–v10), are mostly expressed in cancer cells. The involvement of CD44v, especially CD44v4–v7 and CD44v6–v9, in tumor progression, has been reported in multiple clinical studies. In contrast, other studies have reported that CD44 suppresses breast and prostate cancers [6, 7] and found no correlation between levels of CD44 expression and cancer progression [8]. CD44 can also react with other molecules, including collagen, fibronectin, osteopontin, growth factors and matrix metalloproteinases (MMPs), but the functional importance of these interactions is less well known [1]. It has also been reported that CD44 is involved in intracellular signaling through interactions with neighboring receptors, such as tyrosine kinase receptors, in many types of cancers [9, 10]. Moreover, cleaved CD44 intracellular domain translocates into the nucleus and acts as a signaling molecule [11].

Recently, CD44 has been extensively used as a surface marker to isolate cancer stem cells (CSCs) from breast, prostate, pancreas, ovarian and colorectal cancers [12]. In combination with other surface markers, CD44 can also discriminate between a variety of cancer subsets [13]. CD44-positive cells promote tumorigenesis in breast and colorectal cancers, displaying stem cell properties, such as self-renewal and differentiation. However, the correlation of CD44 expression levels with cancer prognosis and the utility of CD44 as a CSC marker are debatable [14–16]. Differential levels of CD44 expression may account for these discrepancies in the literature and contribute to the ambiguity regarding the functional aspects of CD44. Moreover, there is a lack of clarity regarding the function of CD44 in CSC maintenance and the mechanisms underlying its crosstalk with stemness genes.

Therefore, we undertook this study to detect the ability of CD44 expression to induce mammosphere formation. The mammosphere formation assay can enrich the population of cancer stem cells [17]. Specially, we focused on the function of the CD44 intracellular domain (CD44ICD) with regards to breast cancer stemness. Additionally, we identified the molecular mechanism whereby CD44ICD regulates stemness factors such as Nanog, Sox2, and Oct4, to maintain CSCs and to contribute to the tumorigenesis of breast cancer.

## RESULTS

### Detection of CD44 expression in six breast cancer cell lines

**Figure 1:**
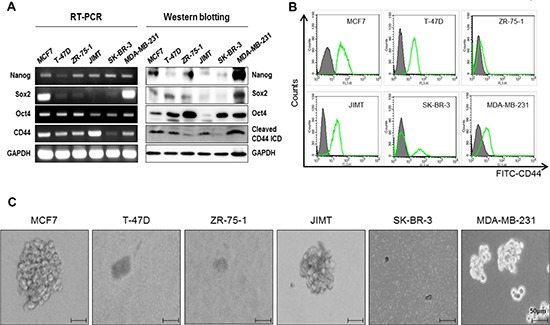
Basal expression levels of CD44 in breast cancer cell lines and mammosphere formation **(A)** The basal expression levels of Nanog, Sox2, Oct4, and CD44 were detected with an RT-PCR (left panel) and western blot (right panel) analysis, respectively. GAPDH was used as a loading control. **(B)** CD44 levels were measured using a FACs analysis. **(C)** The mammosphere forming ability was measured under sphere forming conditions for 15 days as described in “materials methods”. All experiments were performed in the indicated breast cancer cell lines.

We measured the mRNA levels of CD44 and 17 kDa cleaved CD44ICD respectively by RT-PCR and western blot analysis (Figure 1A), as well as by evaluating their surface expression using a FACS analysis in six breast cancer cell lines (Figure 1B). Whereas the CD44 surface expression was detected in five cell lines but not in ZR-75–1 cells, among the six breast cancer cell lines, cleaved CD44ICD was strongly detected in the MCF-7, T47D, JIMT and MDA-MB-231 cell lines. We also measured the expression levels of stemness factors, such as Nanog, Sox2, and Oct4, in these breast cancer cell lines (Figure 1A). The expression of Nanog and Oct4 was not different between cells, whereas Sox2 was highly expressed in MCF-7 and MDA-MB231 cells. We then analyzed the mammosphere formation ability in six cell lines (Figure 1C). Interestingly, mammospheres were readily formed by MCF-7, JIMT, and MDA-MB-231 cells, suggesting that there is a correlation of the mamosphere formation with the expression level of cleaved CD44ICD, rather than with CD44 surface expression.

### Effect of overexpression of CD44ICD in the absence of CD44 on the potential of breast cancer cells for mammosphere formation

**Figure 2:**
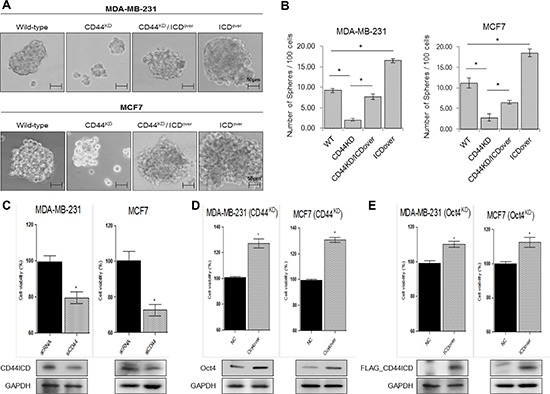
Overexpression of CD44ICD in the absence of CD44 increases the ability of mamosphere formation in breast cancer cells **(A)** The mammosphere-forming ability of the indicated stable lines was measured under sphere forming conditions for 15 days. **(B)** The number of spheres was quantified in the experiments (A) **(C)** Cells were transfected with scRNA and CD44 siRNA. Following transfection, the cells were incubated for 48 hr, and cell proliferation was detected with a WST assay. **(D)** and **(E)** Cells were transfected with control and Oct4 expression vectors (D), and with control and Oct4 expression vectors (E) Following transfection, the cells were incubated for 48 hr, and the cell proliferation detected with a WST assay. The data are presented as the mean ± SD (*n* = 3). Significant differences are indicated by an asterisk (*p < 0.05), and the *p* values were calculated using the Student's *t* test.

To test whether CD44ICD regulates the potential of breast cancer cells for mammosphere formation, we selected MDA-MB-231 and MCF-7 cells and generated stable cell lines, including CD44-depleted cells (CD44^KD^), CD44-depleted cells with overexpression of CD44ICD (CD44^KD^/ICD^over^), and wild-type cells with overexpression of CD44ICD (ICD^over^), using a lentiviral vector with a genotype that was confirmed by RT-PCR (upper panel) and western blotting (lower panel) (Supplementary Figure S1). These cells were mammosphere-cultured for 15 days (Figure 2A and 2B). The size and number of mammospheres were significantly reduced in CD44-depleted MDA-MB-231 and MCF-7 cells. However, CD44-depleted and CD44ICD-overexpressed cells showed an increased size and number of mammospheres. Additionally, the size and number of spheres were greater in cells with CD44ICD overexpression than in wild-type (WT) cells. Treatment with GSI also significantly inhibited the formation of mammospheres in MCF-7 cells (Supplementary Figure S2). We also determined that the cell proliferation of breast cancer MDA-MB231 and MCF-7 cells was significantly reduced by CD44 ablation (Figure 2C). Taken together, our results suggest that CD44ICD might regulate mammosphere formation in breast cancer cells.

### Effect of CD44 on the expression of the stemness factors, Nanog, Sox2, and Oct4

**Figure 3:**
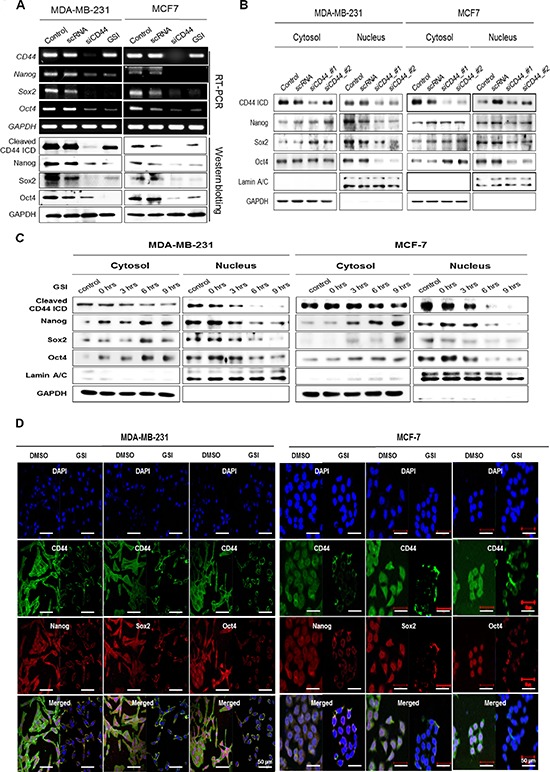
CD44-depletion reduces both the expression and nuclear localization of the stemness factors, Nanog, Sox2, and Oct4 MDA-MB-231 and MCF-7 cells were transfected with scrambled siRNA (scRNA) and CD44 siRNA, and then treated with 5 μM of GSI for 24 hr. **(A)** mRNA (upper) and protein levels (lower) of CD44 and stemness factors were detected with an RT-PCR and western blot analysis. **(B and C)** the changes in the localization of cleaved CD44ICD and stemness factors were detected using western blot analysis. The cells were treated with 5 μM of GSI for the indicated times. GAPDH and Lamin A/C were used as loading controls. **(D)** The changes in the localization of CD44ICD and its co-localization with stemness factors were detected during treatment with 2 μM of GSI for 12 hr in MDA-MB-231 and MCF-7 cells using an immunocytochemical analysis as described in “materials methods”.

To measure the expression levels of Nanog, Sox2 and Oct4 in the absence of CD44, we first obtained three kinds of shRNA for CD44 and confirmed their complete silencing effect on CD44 in both MDA-MB-231 and MCF-7 cells (Supplementary Figure S3). Compared to the expression in control or scRNA transfected cells, we observed down-regulation of the mRNA and protein levels of Nanog, Sox2, and Oct4 in MDA-MB-231 and MCF-7 cells that had undergone CD44 knockdown by CD44 siRNA (Figure 3A). Moreover, treatment with a GSI, which blocks the cleavage of CD44ICD [18], did not change CD44 mRNA expression levels and reduced the cleaved CD44ICD protein levels. It also reduced the mRNA and protein levels of Nanog, Sox2, and Oct4. Moreover, the proliferation of CD44-ablated MDA-MB-231 and MCF-7 cells was rescued by Oct4 overexpression (Figure 2D). The proliferation of Oct4-ablated cells was also rescued by CD44ICD overexpression (Figure 2E) in both MDA-MB-231 and MCF-7 cells. These results suggest that the cleavage of the intracellular domain of CD44 might influence the expression of stemness factors to maintain breast cancer stem cells.

### Effect of the nuclear-translocation of cleaved CD44ICD on the expression and nuclear-localization of Nanog, Sox2, and Oct4

We studied the localization of Nanog, Sox2, and Oct4 by preparing cytosolic and nuclear fractions of CD44-depleted MDA-MB-231 and MCF-7 cells (Figure 3B). In CD44-depleted MDA-MB-231 and MCF-7 cells, the expression of these stemness factors in the cytosolic fraction was similar to or greater than that observed in control cells treated with scrambled RNA. However, the expression of these factors was remarkably reduced in the nuclear fraction, as was the expression of cleaved CD44ICD (Figure 3B). These findings suggest that CD44 depletion inhibits the nuclear localization of Nanog, Sox2, and Oct4. We also blocked the nuclear translocation of CD44ICD using GSI treatment and detected the nuclear localization of stemness factors (Figure 3C). Following GSI treatment, both cytosolic and nuclear CD44ICD expression were significantly decreased in a time-dependent manner in both MDA-MB-231 cells and MCF-7 cells, suggesting that GSI treatment blocked the cleavage and nuclear translocation of CD44ICD. Interestingly, the expression of Nanog, Sox2 and Oct4 in the nuclear fraction decreased with GSI treatment, whereas their expression in the cytosolic fraction increased, suggesting that GSI treatment blocked the nuclear translocation of stemness factors as well as CD44ICD.

The effect of GSI treatment on the localization of the stemness factors was visualized with an immunocytochemical analysis in both MDA-MB-231 and MCF-7 cells (Figure 3D). In the absence of GSI, the expression of CD44ICD and the stemness factors was detected in both the nucleus and cytosol. Following GSI treatment, Nanog, Sox2, and Oct4 were significantly restricted to the cytosolic membrane, similar to CD44ICD localization. Taken together, these data suggest that the nuclear translocation of cleaved CD44ICD is critical to the nuclear localization of the stemness factors, Nanog, Sox2, and Oct4.

### Effect of overexpression of CD44ICD in the absence of CD44 on the expression and nuclear localization of Nanog, Sox2, and Oct4

**Figure 4:**
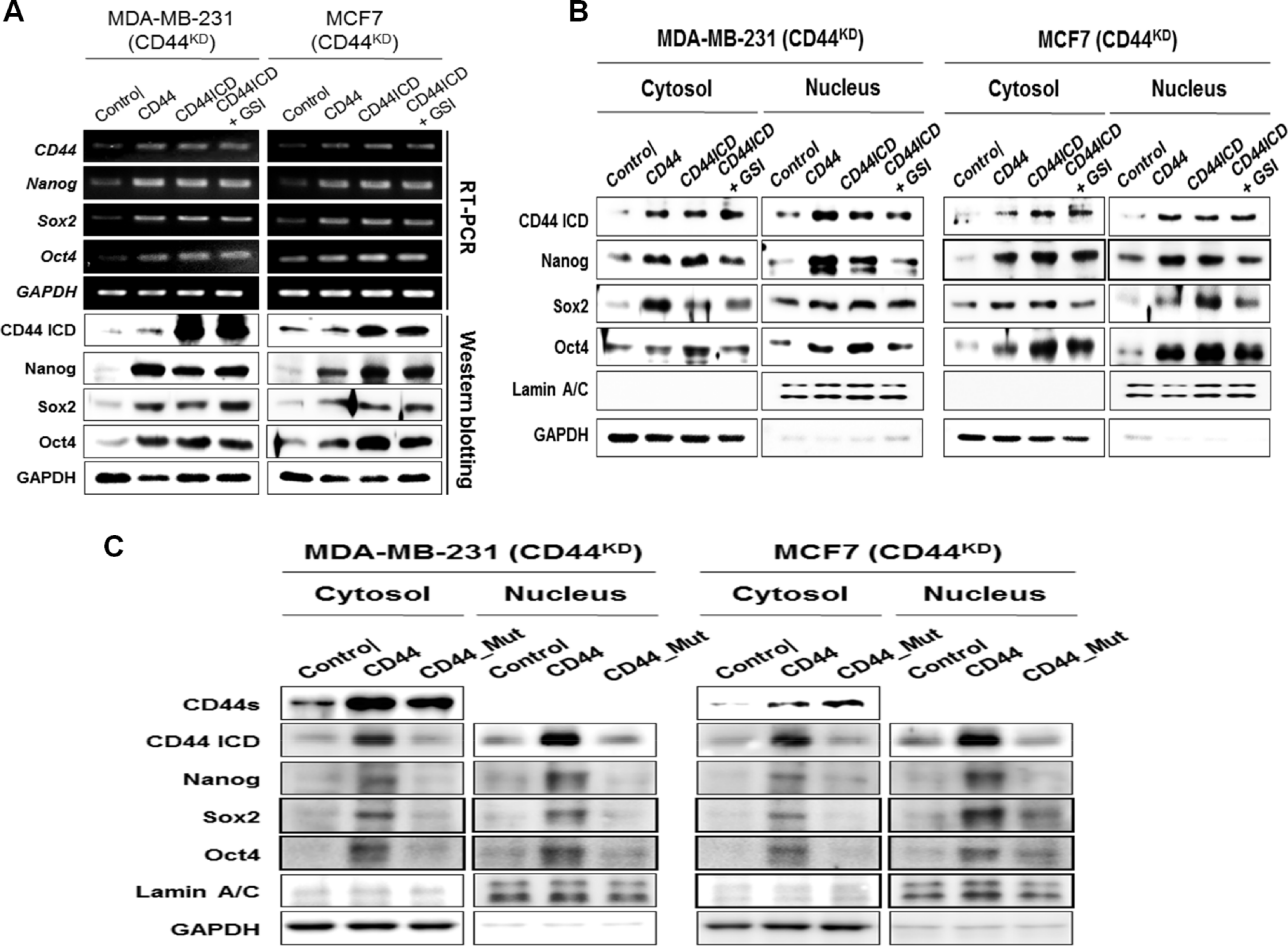
Overexpression of CD44ICD in CD44-depleted cells increases both the expression and nuclear-localization of the stemness factors, Nanog, Sox2, and Oct4 Endogenous CD44 stable knockdown MDA-MB-231 and MCF-7 cells were transfected with the control vector and with the full-length CD44 and CD44ICD expression vectors. CD44ICD transfected cells were treated with 5 μM of GSI for 24 hr. **(A)** The mRNA (upper) and protein levels (lower) of CD44 and stemness factors were detected with an RT-PCR and western blot analysis. **(B)** The changes in the localization of CD44ICD and stemness factors were detected with a western blot analysis. GAPDH and Lamin A/C were used as loading controls. **(C)** Endogenous CD44 stable knockdown MDA-MB-231 and MCF-7 cells were transfected with the control and full-length form of CD44, and with the cleavage site truncated mutant CD44 vector. The changes in the localization of CD44ICD and stemness factors were detected with a western blot analysis.

The overexpression of CD44ICD in CD44-depleted cells significantly increase the mRNA and protein expression levels of the stemness factors, which were similar to those in the cells with overexpression of full-length CD44 (Figure 4A). To detect the effect on another molecules by GSI, because GSI also blocks cleavage of other molecules, such as Notch and ErB-4 [19], we evaluated the effect of GSI on the nuclear localization of these stemness factors following CD44ICD overexpression (Figure 4A). Whereas GSI reduced the expression of these stemness factors, GSI did not affect the expression of the same stemness factors following overexpression of CD44ICD, suggesting that the GSI effect on the expression of stemness factor is dependent on CD44ICD.

The localization of Nanog, Sox2, and Oct4 was also evaluated in the context of overexpression of CD44ICD in CD44-depleted MDA-MB-231 and MCF-7 cells (Figure 4B). Their expression levels were primarily reduced in the nuclear fraction of CD44-depleted cells, whereas they were increased in both the cytosolic and nuclear fractions by the overexpression of full-length CD44 or the overexpression of CD44ICD in CD44-depleted cells. As expected, GSI did not influence the localization of the stemness factors in CD44ICD-overexpressed and CD44-depleted cells. The increased nuclear accumulation of Nanog, Sox2, and Oct4 following the overexpression of CD44ICD was visually confirmed using immunocytochemistry (Supplementary Figure S4). The overexpression of CD44ICD strongly promoted the nuclear localization of the stemness factors and their co-localization with CD44ICD. These results suggest that the nuclear localization of Nanog, Sox2, and Oct4 is regulated by CD44ICD.

We further prepared the cleavage site truncated mutant CD44 (CD44_Mut), which cannot release cleaved CD44ICD, and overexpressed it in MDA-MB-231 and MCF-7 cells to detect its influence on the nuclear localization of the stemness factors (Figure 4C). Whereas the overexpression of CD44 induced the expression of these stemness factors in both the cytosol and nucleus, the overexpression of CD44_Mut did not significantly influence the expression of the stemness factors in either the nucleus or the cytosol fractions. Taken together, these results suggest that the cleavage and nuclear translocation of CD44ICD is critical for the nuclear localization of the stemness factors.

### Effect of the C-terminal of CD44ICD on its interaction with Nanog, Sox2, and Oct4

**Figure 5:**
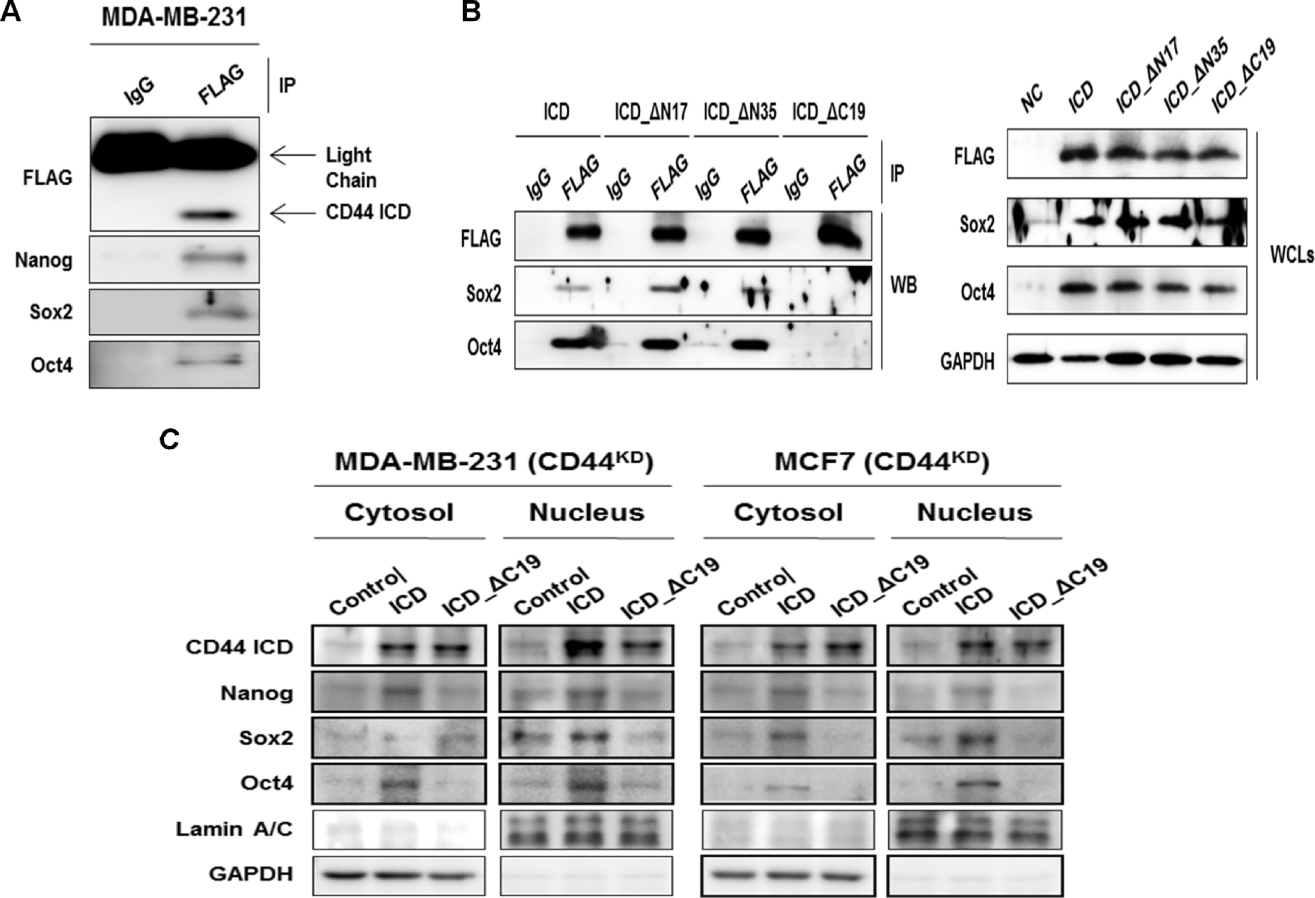
CD44ICD interacts with the stemness factors, Nanog, Sox2, and Oct4 and regulates the nuclear-localization **(A)** MDA-MB-231 cells were transfected with CD44ICD. The interaction between CD44 and Nanog, Sox2, and Oct4 were detected with an immunoprecipitation assay described in “materials and methods”. **(B)** HEK293 cells were transfected with CD44ICD or the truncated mutant constructs, ICD_ΔN17, ICD_ΔN35, ICD_ΔC19, and co-transfected with Sox2 and Oct4 expression vectors for 36 hr as described in “materials methods”. Their interaction was detected with an immunoprecipitation assay. **(C)** Endogenous CD44 stable knockdown MDA-MB-231 and MCF-7 cells were transfected with the control and CD44ICD and C-terminal region truncated CD44 vectors. The changes in the localization of CD44ICD and stemness factors were detected by western blot. GAPDH and Lamin A/C were used as loading controls.

Next, we performed an immunoprecipitation assay and investigated whether CD44ICD interacted directly with Nanog, Sox2, and Oct4 using MDA-MB-231 cell lysates (Figure 5A). We generated an N-terminal truncated mutant CD44-ICD expressing vector (ICD_ΔN35), and a NLS truncated mutant construct, CD44-ICD_ΔN17, because the NLS of CD44ICD is located in the N-terminal region [20] and the C-terminal truncated mutant CD44ICD_ΔC19, as indicated in Figure 5B. We then, co-transfected these mutant vectors and Sox2- or Oct4-expressing vectors to detect their interaction site. The C-terminal truncated mutant CD44ICD_ΔC19 did not interact with Sox2 and Oct4 (Figure 5B). This suggests that the essential region for the interaction with Sox2 and Oct4 is located in the C-terminal region of CD44ICD, including the PDZ domain [5]. We overexpressed the C-terminal truncated mutant CD44IC_ΔC19 and fractionated the nucleus and cytosol (Figure 5C). As expected, the reduced nuclear localization of both CD44ICD and the stemness factors following the overexpression of the C-terminal truncated mutant CD44IC_ΔC19 was detected in MDA-MB-231 and MCF-7 cells (Figure 5C). Taken together, our results suggest that CD44ICD regulates the nuclear localization and transcriptional activation of stemness factors through an interaction with its C-terminal domain.

### Effect of overexpression of CD44ICD in the absence of CD44 on the transcriptional activation of Sox2 and Oct4

**Figure 6:**
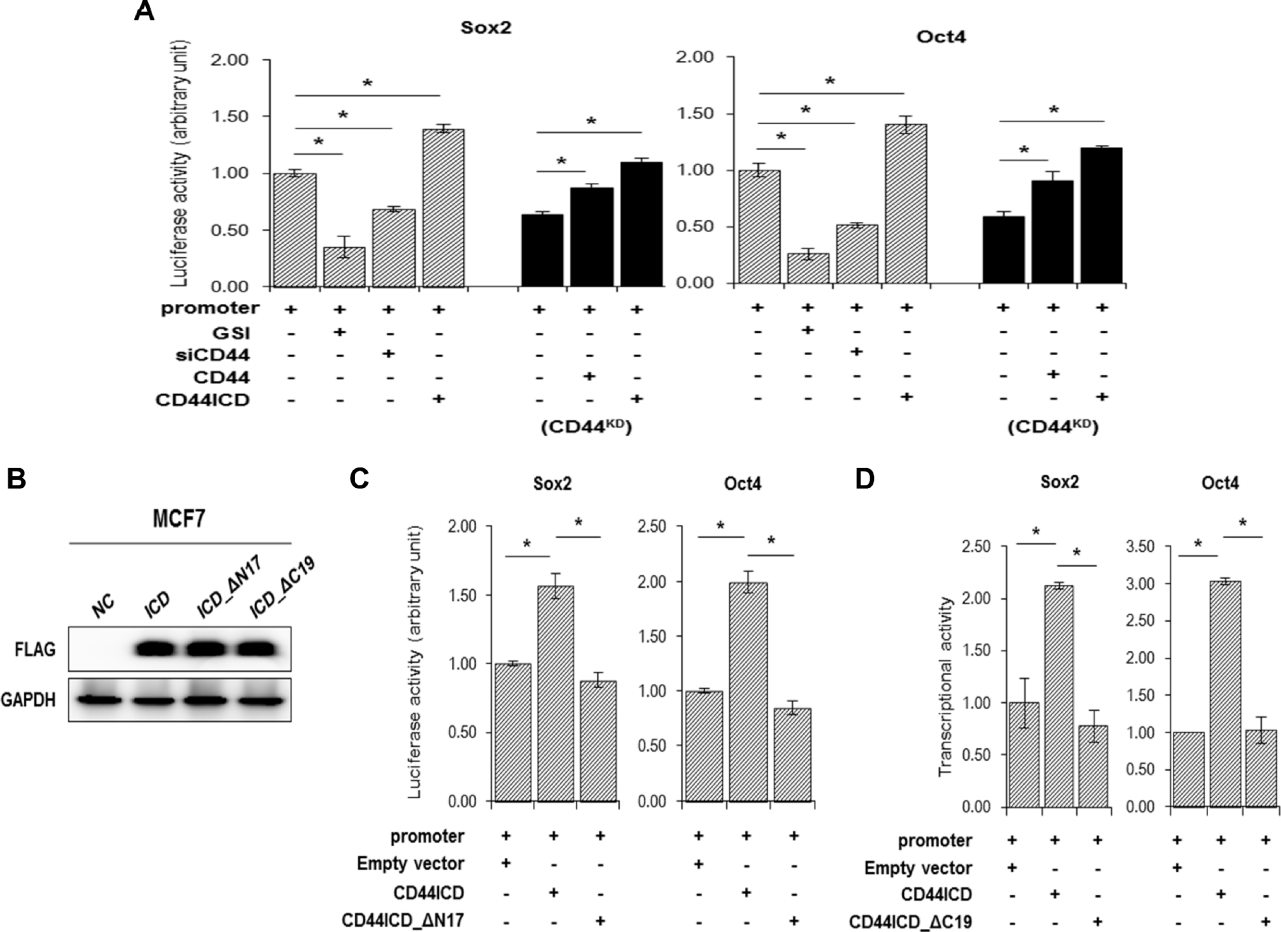
Overexpression of CD44ICD increases the transcriptional activation of the stemness factors, Sox2, and Oct4 **(A)** The transcriptional activation was measured with a reporter assay in wild-type (diagonal bars) and CD44KD MCF-7 (solid bars) cells. Cells were transfected with a Sox2 and Oct4 reporter vector alone or co-transfected with CD44 siRNA, CD44 or CD44ICD expression vectors. Following transfection, the cells were incubated for 12 hr and a vehicle (−) or 5 μM of GSI (+) were added. The cells were incubated for an additional 24 hr. The transcriptional activity was measured by luciferase activity described in “materials and methods”. **(B)** The expression of CD44ICD or of the truncated mutant constructs, ICD_ΔN17 and ICD_ΔC19 were detected with a western blot. **(C)** MCF-7 cells were transfected with the reporter vector alone or co-transfected with CD44-ICD or CD44-ICD_ΔN17 expression vectors for 36 hr. The luciferase activity was measured as described in “materials methods”. **(D)** MCF-7 cells were transfected with the reporter vector alone or co-transfected with CD44ICD or CD44ICD_ΔC19 expression vectors for 36 hr. The luciferase activity was measured. The data are presented as the mean ± SD (*n* = 3). Significant differences are indicated by an asterisk (*p < 0.05), and the *p* values were calculated using the Student's *t* test.

We measured the luciferase activity of the Sox2 and Oct4 promoter reporters to check whether CD44ICD also regulates the transcriptional activation of Sox2 and Oct4 (Figure 6A). In control cells, transcriptional activation of the Sox2 promoter (left graph) and the Oct4 promoter (right graph) was repressed by both GSI and CD44 siRNA treatment (diagonal bars in Figure 5A). The overexpression of CD44ICD enhanced the transcriptional activation of the Sox2 and Oct4 promoters more than in control cells. In CD44-depleted stable cells, the overexpression of CD44ICD alone induced the transcriptional activation of Sox2 and Oct4 more than in cells that overexpressed the full-length form of CD44 (solid bars in Figure 5A).

We also over-expressed CD44-ICD_ΔN17 and the C-terminal truncated mutant CD44IC_ΔC19 (Figure 6B) and measured the luciferase activity of the Sox2 and Oct4 promoters (Figure 6C and 6D). Interestingly, the transcriptional activation of the Sox2 promoter (left graph) and the Oct4 promoter (right graph) was not influenced by transfection with CD44ICD_ΔN17 (Figure 6C). In addition, the transcriptional activation of the Sox2 and Oct4 promoters was not enhanced when transfected with CD44ICD_ΔC19 compared to the transcriptional activation in the cells that overexpressed CD44ICD (Figure 6D). It suggests that the NLS region of CD44ICD for the nuclear localization of CD44 and the interaction between CD44ICD and stemness factors are important to the transcriptional activation of Sox2 and Oct4.

### Effect of overexpression of CD44ICD in the absence of CD44 on breast cancer tumorigenesis and metastasis *in vivo*

**Figure 7:**
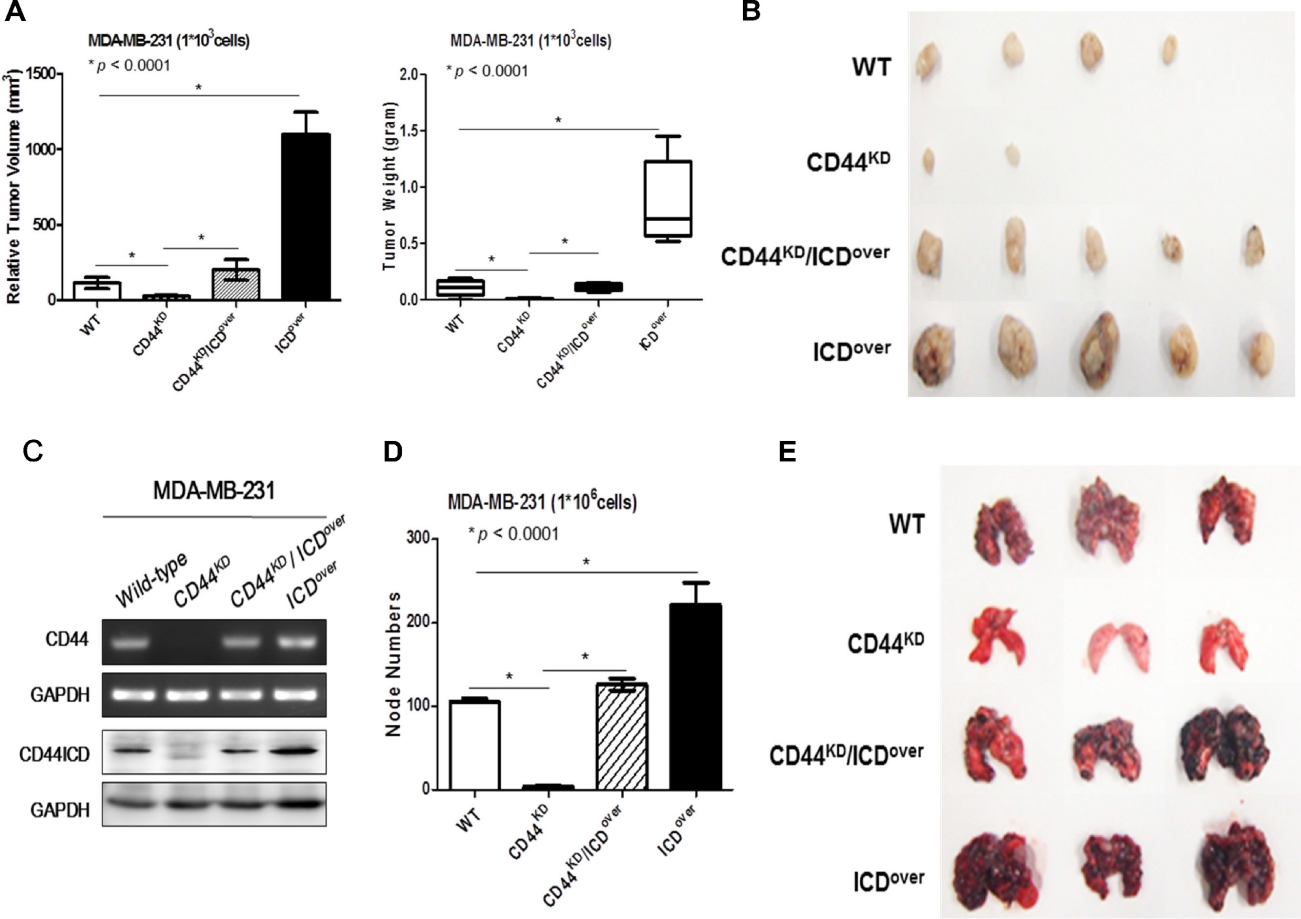
Overexpression of CD44ICD in CD44-depleted cells increased tumorigenesis and metastasis in breast cancer cells in xenograft mice *in vivo* **(A and B)** The indicated stable lines of MDA-MB-231 cells (1 × 103) were implanted into nude mice to form subcutaneous xenografts, as described in “materials methods”. After 21 days, the tumors were isolated and the size and weight of those measured are presented in a statistical graph (A) and in a photograph (B). **(C)** The knockdown of CD44 and overexpression of CD44ICD were confirmed with an RT-PCR (upper panel) and western blot analysis (lower panel) in MDA-MB-231 cells. **(D and E)** The indicated stable lines of MDA-MB-231 cells (1 × 106) were injected into the tail veins of nude mice, as described in “materials methods”. After 21 days, the lungs were isolated and the node numbers were counted; they are presented in a statistical graph (D) and in a photograph (E). The data are presented as the mean ± SD (*n* = 5). A statistical analysis was performed using a one-way ANOVA, and significant differences are indicated by an asterisk (*p < 0.0001).

To analyze the *in vivo* effect of CD44ICD on breast cancer tumorigenesis, we prepared stable cell lines (Figure 7C) and xenografted mice using the following cell types as previously used: CD44^KD^, CD44^KD^/ICD^over^ and ICD^over^ (Figure 7A and 7B). Whereas the xenografts with CD44-depleted MDA-MB-231 cells did not form tumors, the largest tumor burdens were detected in MDA-MB-231 cells with CD44ICD overexpression. The overexpression of CD44ICD in CD44-depleted MDA-MB-231 cells also promoted tumor growth in xenografted mice as well as in control cells. Furthermore, the CD44-depleted stable cells did not form tumors in additional xenografts using different cell numbers (Table 1).

**Table 1:** Overexpression of CD44ICD in CD44-depleted MDA-MB-231 cells increases lung metastasis in mice The indicated stable lines of MDA-MB-231 cells (1 × 10^6^) were injected into the tail veins of nude mice as described in “material and methods”. After 21 days, the lungs were isolated and the number of mice with tumor node bearing lungs was counted.

MDA-MB-231	Tumorigenesis			Metastasis
	10^3^ Cells	10^4^ Cells	10^5^ Cells	10^6^ Cells
Wild Type	4/5	5/5	5/5	4/5
CD44^KD^	2/5	2/5	3/5	0/5
CD44^KD^ / ICD^over^	5/5	5/5	5/5	4/5
CD44 ICD^over^	5/5	5/5	5/5	5/5

The cells were derived from spheres

We also injected the cells into the tail veins of mice, and detected colonies of tumor cells in their lungs (Figure 7D and 7E). While we rarely detected colonies of CD44-depleted MDA-MB-231 cells in mice lungs, colonies of MDA-MB-231 cells with CD44ICD overexpression were detected frequently in mouse lungs. Colonies of MDA-MB-231 cells with CD44ICD overexpression and CD44-depletion were detected more frequently than colonies of CD44-depleted cells. These results show that the overexpression of CD44ICD strongly accelerates tumor progression and metastasis in mice *in vivo*, a similar finding to our *in vitro* results.

## DISCUSSION

In this study, we observed that the expression of cleaved CD44ICD in breast cancer cells is an active regulator of mammosphere formation. We used a serum-free non-adherent culture technique, as used in previous studies [21]. whereas most cells died after being plated onto the non-adherent surface, those that survived formed spherical colonies termed mammospheres and the enriched cells were shown to be stem cells. This suggested that the cleavage of CD44ICD potentially regulates cancer stemness characteristics, which led us to investigate the γ-secretase-dependent cleavage of CD44ICD. Several substrates for the presenilin-dependent γ-secretase have recently been identified, including Notch, E-cadherin, ErbB-4, and a β-amyloid precursor protein [19]. This cleavage releases CD44ICD and allows its translocation into the nucleus. Nuclear CD44ICD regulates transcriptional activation through several transcriptional factors, such as CBP/p300 and STAT3 [22]. There is also evidence that the full-length form of CD44 translocates into the nucleus and activates STAT3 [23]. Therefore, we overexpressed CD44ICD alone in CD44-depleted cells. The overexpression of CD44ICD enhanced both mammosphere formation and the expression of the stemness factors, Nanog, Sox2 and Oct4. The overexpression of CD44ICD containing a mutant NLS did not have these effects, suggesting that in addition to the requirement the full length form of CD44, the cleavage and nuclear translocation of CD44ICD is also important for the expression and activation of stemness factors.

Coordinated networks of stemness factors are the master regulatory mechanisms of pluripotency and differentiation in stem cells. Stem cell-specific transcription factors, such as Nanog, Sox2 and Oct4 alone and in combination have been studied in embryonic stem (ES) cell pluripotency and cancer stem cell formation [24]. For example, Nanog overexpression enables the propagation of mouse ES cells [25]. Although Nanog is not required for the establishment of pluripotency in ES cells, it does maintain the self-renewal capacity of these cells, and its expression has been shown to suppress differentiation. Sox2 is a member of the SRY-related HMG box (Sox) transcription factor family [26]. Sox2 remains less well characterized than either Oct4 or Nanog, but Sox2 is known to play a major role in the regulation of stem cell fate. Moreover, the down-regulation or overexpression of Oct4 leads to a loss of pluripotency in ES cells as the cells differentiate [27, 28]. This suggests that precise levels of Oct4 are required for the maintenance of stem cell pluripotency. Additionally, it is clear that these stemness factors function collaboratively to regulate the state of differentiation of ES cells. Sox2 and Oct4 are known to act synergistically to regulate their own transcription as well as the expression of other key stem cell genes, including *NANOG* [2]. These stemness factors also co-occupy genes and share a substantial fraction of target genes. About half of the promoter regions bound by Oct4 has been shown to also be bound by Sox2 and Nanog [29]. Moreover, the binding sites of Nanog, Sox2 and Oct4 are in close proximity to each other, further confirming that the proteins work in concert.

We also found that CD44ICD shares characteristics with Nanog, Sox2, and Oct4. Evidence of a direct interaction between the C-terminal region of CD44ICD and these stemness factors, and of the coordination of their nuclear translocation was observed. Previously, it was reported that HA stimulates the interaction of CD44v3 interaction with Oct4-Sox2-Nanog, leading to complex formation and nuclear translocation [30]. The interaction between HA and CD44 mediates Nanog-Stat-3 signaling pathways that induce *MDR1* expression and ankyrin/cytoskeleton functions [31]. Others have investigated the interaction between CD44 and stemness factors and have suggested that HA regulates the CD44-mediated activation of stemness factors. We found the first piece of evidence of a direct interaction between the C-terminal region of CD44ICD and stemness factor transcription and their co-translocation into the nucleus. In our study, the overexpression of CD44ICD itself induced the nuclear translocation and transcriptional activation of stemness factors, suggesting that the activation of stemness factors and the maintenance of stemness by CD44ICD occurs in an HA-independent manner. However, more studies are needed to elucidate the HA-induced cleavage of CD44ICD and the interaction between CD44ICD and stemness factors, which might be important in tumor microenvironments.

Our results suggest that the expression of CD44 is important to the maintenance and possible acceleration of tumorigenesis through the cleavage of its intracellular domain and its interaction with stemness factors. CD44 has already been targeted in breast cancer therapy [2] using monoclonal antibodies [32] and HA-tagged drugs [33]. We propose that GSI shows potential in being used as an inhibitor of the cleavage of CD44ICD. To be used as a drug targeted to cancer stem cells, GSI would require an enhanced specificity to CD44ICD or to the interaction between CD44ICD and stemness factors, or both.

## MATERIALS AND METHODS

### Cell culture and transfection

The human breast cancer cell lines, MCF-7, T-47D, ZR-75–1, JIMT, SK-BR-3 and MDA-MB-231, were obtained from the ATCC and maintained in RPMI 1640 and Dulbecco's Modified Eagle Medium (DMEM) with 10% fetal bovine serum (FBS) and 1% antibiotics (Invitrogen, San Diego, CA). Cells cultures were maintained at 37°C in an atmosphere of 5% CO_2_, as previously described [34]. Transfection with CD44, CD44ICD, the truncated mutant CD44ICD, Sox2, and Oct4 expression vectors [35] as well as with CD44 siRNA was performed with Lipofectamine 2000 and Lipofectamine RNAiMAX reagents (Invitrogen), according to the reagent manufacturer's instruction. CD44 siRNA #1 (5′-UAUUCAAAUCGAUCUGCGCUU-3′) and CD44 siRNA #2 (5′-GACCAAUUACCAUAACUAUU-3′) were purchased from Genolution. Cells were harvested two days after transfection for use in the experiments.

### Mammosphere culture

Cells were grown in ultra-low attachment plates (Corning) and in Mammary Epithelium Basal Medium (MEBM; Lonza) supplemented with B27 (Gibco), 20 ng/ml of EGF, and 20 ng/ml of bFGF (PeproTech) at a density of 1000 cells/ml. After culturing the cells for 15 days, we counted the mammospheres with diameters > 50 μm.

### Cloning and generation of stable cell lines

The CD44 coding region (GenBank accession number NM_000610.3) was isolated and cloned into the pLECE3 lentiviral vector with an N-terminal Flag-tag [36]. PCR analysis of cDNA was used to generate expression constructs of full-length CD44; CD44-ICD; and mutant constructs with a 17 amino acid truncation at the N-terminal (CD44ICD_ΔN17), 35 amino acid truncation at the N-terminal (CD44ICD_ΔN35), and 19 amino acid truncation at the C-terminal (CD44ICD_ΔC19), respectively, and an essential four amino acid (I^287^ ~ N^290^) truncation at the cleavage site (CD44_Mut) [11]. The primers are described in Supplementary Table S1. The PCR analysis was performed following the Ex-Taq manual (TaKaRa, Shiga, Japan). For subcloning, two restriction enzyme sites, Pac1 and Not1 (NEB), were incorporated into the primers and are underlined in Supplementary Table S1. The shRNA-expressing lentiviral vectors for the CD44 and Oct4 knockdown cells targeted 3′UTR and were purchased from Sigma. Lentivirus production and the generation of stable cell lines have been previously described [37].

### Total RNA isolation and reverse transcriptase-polymerase chain reaction

RNA isolation was performed with the TRIzol^®^ Reagent (Invitrogen) according to the manufacturer's instructions. Subsequently, we performed a reverse transcription PCR using a reverse transcription system (Promega). The primers are described in Supplementary Table S1. The PCR was performed following the Ex-Taq manual.

### Luciferase assay

For the Sox2 and Oct4 reporter assays, Sox2 and Oct4 promoter constructs, pGL3-SRR2 [38] and phOct4-Luc [35], were used. MCF-7 cells were transfected with the plasmids and a β-galactosidase expression vector for normalization. After 48 hr, the luciferase activity was measured using a luciferase assay system (Promega) according to the manufacturer;s instruction.

### Fractionation of cellular extracts and western blotting

Nucleic and cytosolic extracts were prepared from MDA-MB-231 and MCF-7 cells following CD44 siRNA transfection and GSI treatment and from CD44^KD^ MDA-MB-231 and MCF-7 cells following CD44 or CD44-ICD overexpression, as described previously [39].

For western blotting, the cells were lysed in a RIPA buffer (Biosesang) containing a protease inhibitor cocktail (Sigma). The primary antibodies were anti-GAPDH, anti-CD44, anti-Nanog, anti-Sox2, and anti-Oct4 and were purchased from Santa Cruz, as well as anti-FLAG, which was purchased from Sigma. The proteins of interest were detected using ECL solutions (Amersham Life Science) with a LAS-3000 (Fujifilm) detector according to the manufacturer's directions.

### Immunoprecipitation

Cell lysates were pre-cleared by being incubated with 20 μl of protein A/G-linked agarose beads (Santa Cruz) for 1 hr at 4°C. After spinning down the beads, the supernatant was incubated with 2 μg of a specific antibody (anti-FLAG, anti-CD44, anti-Nanog, anti-Sox2, and anti-Oct4) overnight at 4°C, followed by incubation with 40 μl of protein A/G-linked agarose beads for 1 hr. A mouse or rabbit IgG (Santa Cruz) was used as the negative control. Following the incubation, the beads were washed three times in a RIPA buffer before being dissolved in a SDS-PAGE loading buffer. A western blot analysis was performed as described elsewhere [40].

### Cell proliferation analysis

Cells were grown in 96-well culture plates and transfected with CD44 siRNA or Oct4 and CD44-ICD expression vectors. After 48 hr, a WST solution (Daeil, Korea) was subsequently added to each well. After 1–3 hr of additional incubation, the absorbance was measured on and ELISA reader at a test wavelength of 450 nm.

### Flow cytometric experiments

For the flow cytometric experiments, cells from the breast cancer cell lines (MCF-7, T47D, ZR-75–1, JIMT, SK-BR-3 and MDA-MB-231) were detached and washed with PBS supplemented with 2% FBS. The following antibody was used: FITC-conjugated anti-human CD44 (BD Pharmingen). Between 30,000 and 50,000 cells were incubated with the antibody for 30 min on ice. Following the wash, the cells were fixed with 4% paraformaldehyde at 4°C prior to analysis. A minimum of 10,000 events per sample were collected using the FACSDiva and Cell Quest applications (BD Biosciences).

### Immunocytochemistry

CD44, Nanog, Sox2, and Oct4 were detected immunocytochemically in MDA-MB-231 and MCF-7 cells after GSI treatment or CD44ICD transfection. The cells were fixed using 3% formaldehyde, blocked with 5% bovine serum albumin in phosphate buffered saline (PBS), and incubated with primary antibodies, including anti-CD44, anti-Nanog, anti-Sox2, and anti-Oct4, diluted (1:200) in PBS. The cells were conjugated with the secondary antibodies labeled with FITC or Cy5 (Invitrogen), diluted (1:200) in PBS. The samples were treated with mounting medium with 4′, 6-diamidino-2-phenylindole and analyzed using an LSM 700 confocal microscope (Carl-Zeiss).

### Animal experiments

All animal experiments were approved by the Institutional Review Board of the Yonsei University College of Medicine and were performed in specific pathogen-free facilities in accordance with the University's Guidelines for the Care and Use of Laboratory Animals (2013–0018). The preparation of xenografted mice was performed as described previously [41], as was the preparation of lung metastasized mice [40].

### Statistical analysis

Two tumors per mouse were obtained and analyzed to obtain the mean tumor volume per mouse. Unpaired *t*-tests were used to analyze the mean tumor volume of the xenografted mice. Statistical analyses were performed using the Student's *t*-test. The data were considered statistically significant for *p*-values of < 0.05. Statistical analyses were performed using the GraphPad Prism software (version 6; GraphPad Software Inc., La Jolla, CA).

## SUPPLEMENTARY FIGURES AND TABLE


