# Supplementary material for: Cleaved CD44 intracellular domain supports activation of stemness factors and promotes tumorigenesis of breast cancer
Source: Oncotarget. 2015 Apr 2;6(11):8709–21. doi: 10.18632/oncotarget.3325 (PMC4496178; doi:10.18632/oncotarget.3325)
Supplement: Supplementary file 1 [file oncotarget-06-8709-s001.pdf]

SUPPLEMENTARY FIGURES AND TABLE

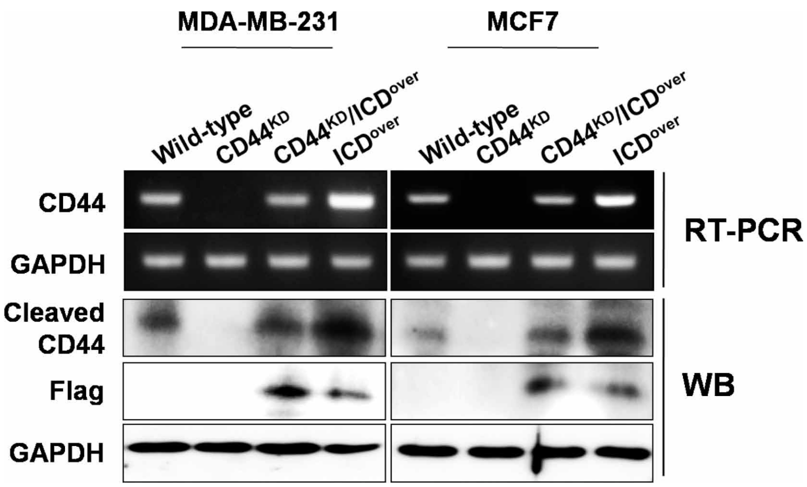

Supplementary Figure S1: Ablation of CD44 and overexpression of CD44ICD by lentiviral vectors. MDA-MB-231 (upper panel) and MCF7 (lower panel) cells were transfected with control and CD44-depleted cells ($CD44^{KD}$), CD44-depleted cells with overexpression of CD44ICD ($CD44^{KD}/ICD^{over}$) and wild-type cells with overexpression of CD44ICD (ICD^{over}) using a lentiviral vector. The mRNA (upper panel) and protein levels (lower panel) of CD44 and Flag were detected by RT-PCR and western blotting, respectively.

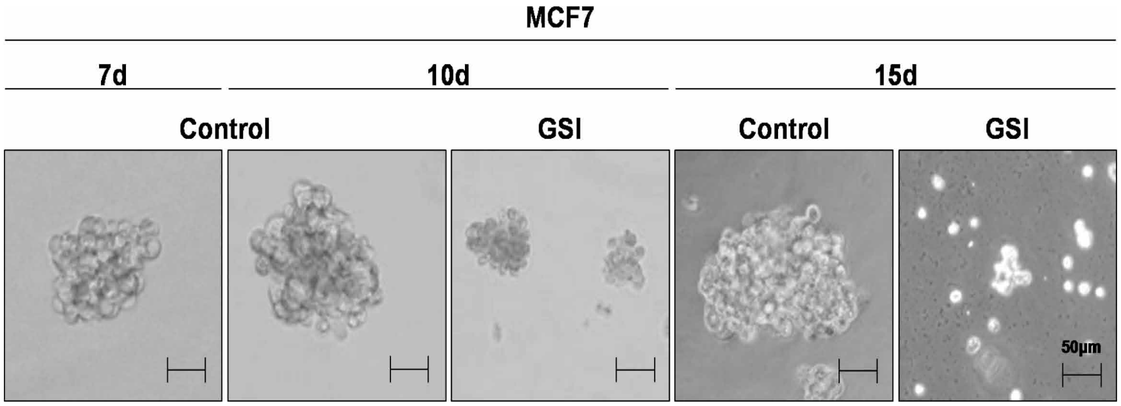

Supplementary Figure S2: Gamma secretase inhibitor as an inhibitor of CD44ICD cleavage prevents sphere formation in breast cancer cells. After 7 days of mammosphere culture, 5 µM of GSI or vehicle were added and the cell culture was continued. Mammosphere forming ability was measured under sphere forming conditions on the indicated days.

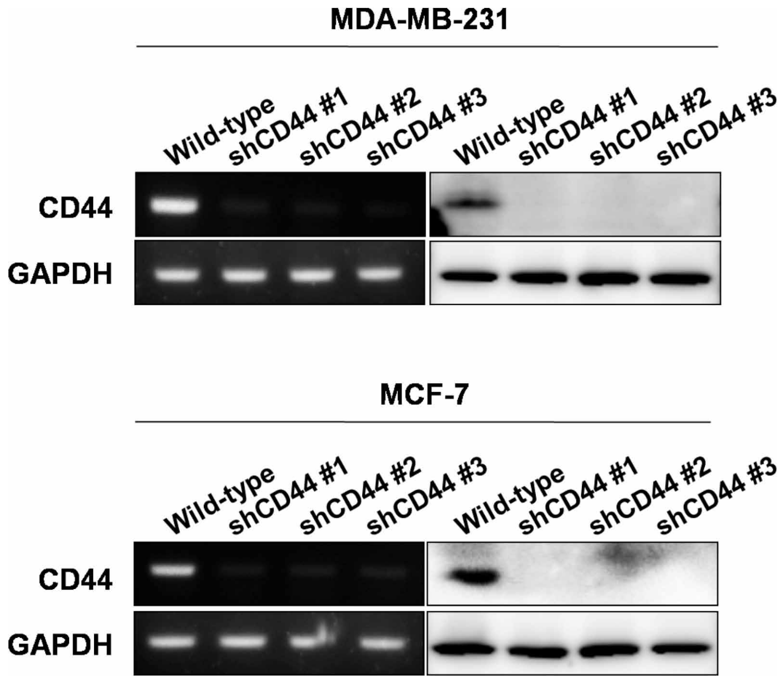

Supplementary Figure S3: Ablation of CD44 by CD44 shRNA. MDA-MB-231 (upper panel) and MCF7 (lower panel) cells were transfected with control and CD44 shRNA lentiviral expression vectors. The mRNA (left panel) and protein levels (right panel) of CD44 were detected by RT-PCR and western blotting, respectively.

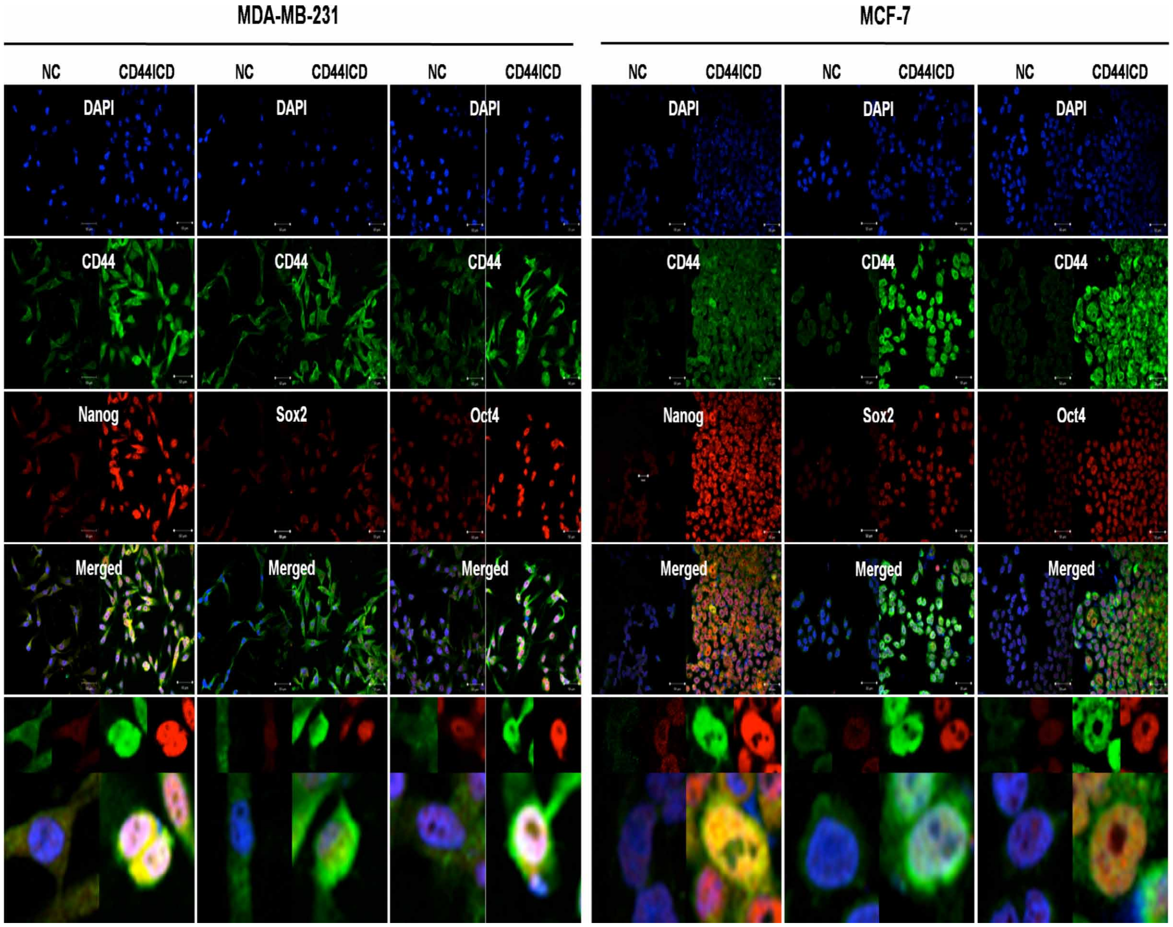

Supplementary Figure S4: Overexpression of CD44ICD induced nuclear localization of stemness factors, Nanog, Sox2, and Oct4. Co-localization of CD44ICD with stemness factors was detected after CD44ICD overexpression using immunocytochemical analysis.

Supplementary Table S1: Primer lists for cloning and RT-PCR

Primer	Sequence (5′ to 3′)	Applications
CD44_For	TTCCCAAAAAGAGGCTGAGA	CD44 RT-PCR
CD44_Rev	CAATGTTGCAAGGGTTTGTG	
Nanog_For	ACCTTCCAATGTGGAGCAAC	Nanog RT-PCR
Nanog_Rev	GAATTTGGCTGGAACTGCAT	
Sox2_For	AAAACAGCCCGGACCGCGTC	Sox2 RT-PCR
Sox2_Rev	CTCGTCGATGAACGGCCGCT	
Oct4_For	CTCACCCTGGGGGTTCTATT	Oct4 RT-PCR
Oct4_Rev	CTGGTTCGCTTTCTCTTTCG	
GAPDH_For	GGCTGCTTTTAACTCTGGTA	GAPDH RT-PCR
GAPDH_Rev	ACTTGATTTTGGAGGGATCT	
CD44_For	GATCTTAATTAACATGGACAAGTTTTGGTGGCAC	CD44 cloning
CD44_Rev	GATCGCGGCCGCTTATTACACCCCAATCTTCATGTCCAC	
CD44ICD_For	GATCTTAATTAACGCAGTCAACAGTCGAAGAAGG	CD44ICD cloning
CD44ICD_ΔN17_For	GATCTTAATTAACAGTGGCAATGGAGCTGTGGAG	CD44ICD_ΔN17 cloning
CD44ICD_ΔN35_For	GATCTTAATTAACAGCAAGTCTCAGGAAATGGTG	CD44ICD_ΔN35 cloning
CD44ICD_ΔC19_Rev	GATCGCGGCCGCTTATTACTGGTCTGGAGTTTCTGACGA	CD44ICD_ΔC19 cloning
CD44ICD_Mut_For	AGTCGAAGAAGGTGTGGGCAG	CD44ICD_Mut cloning
CD44ICD_Mut_Rev	GCAAACTGCAAGAACCAAAGCC	CD44ICD_Mut cloning
